# Tools to predict acute traumatic coagulopathy in the pre-hospital setting: a review of the literature

**DOI:** 10.29045/14784726.2020.09.5.3.23

**Published:** 2020-12-01

**Authors:** Simon Robinson, Jordan Kirton

**Affiliations:** London Ambulance Service; South West Ambulance Service

**Keywords:** acute traumatic coagulopathy, damage control resuscitation, decision tools, major trauma; paramedic, pre-hospital care

## Abstract

**Introduction:**

Recognising acute traumatic coagulopathy (ATC) poses a significant challenge to improving survival in emergency care. Paramedics are in a prime position to identify ATC in pre-hospital major trauma and initiate appropriate coagulopathy management.

**Method:**

A database literature review was conducted using Scopus, CINAHL and MEDLINE.

**Results:**

Two themes were identified from four studies: prediction tools, and point-of-care testing. Prediction tools identified key common ATC markers in the pre-hospital setting, including: systolic blood pressure, reduced Glasgow Coma Score and trauma to the chest, abdomen and pelvis. Point-of-care testing was found to have limited value.

**Conclusion:**

Future research needs to explore paramedics using prediction tools in identifying ATC, which could alert hospitals to prepare for blood products for damage control resuscitation.

## Introduction

Traumatic injury causes approximately 2 million deaths annually and is recognised as the leading cause of preventable mortality in people aged 44 and under (Trauma Audit and Research Network, 2017). It is thought that approximately 33% of major trauma patients develop coagulopathy, 76% of which will have acute traumatic coagulopathy (ATC) prior to hospital arrival (Cohen & Christie, 2017). ATC occurs at the point of trauma, where tissue damage is sufficient that inflammation, fibrinolysis (clot breakdown) and systemic hypoperfusion develop into a failure of the coagulation system to sustain adequate haemostasis (Davenport, 2013). It is thought to have a mortality rate of 50% (Brohi & Eaglestone, 2017).

While paramedics are trained to triage major trauma with the aid of major trauma triage tools (Thompson et al., 2017), identifying major trauma remains a complex process, particularly in patients over the age of 55 years (Durham, 2017). In addition, ATC is not considered in existing pre-hospital triage tools, despite guidance from the National Institute of Health and Care Excellence (NICE) that early recognition of ATC is a key goal of trauma management (NICE, 2016). Timely treatment of ATC is vital in improving patient outcomes (Cohen & Christie, 2017). If paramedics could reliably identify patients at risk of ATC, and implement specific care pathways (such as accelerated blood transfusion activation), this might improve patient outcomes from trauma complicated by ATC.

This literature review aims to identify if pre-hospital screening tools can recognise ATC.

## Method

An integrative literature review methodology was decided as a pragmatic approach in order to explore key themes that might establish future research. Throughout October 2019, the databases MEDLINE and CINAHL (accessed via EBSCOhost) and Scopus were searched for relevant articles. For keywords, a search filter derived by Olaussen et al. (2017) was used, consisting of Ambulances OR Emergency Medical Technicians OR Air Ambulances OR paramedic* OR ems OR emt OR prehospital OR pre-hospital OR first responder* OR emergency medical technicians OR emergency services OR Ambulance* OR HEMS OR field triage. Medical subject headings identified topic-specific keywords for trauma, including: injuries OR wounds and injuries; and coagulopathy.

Inclusion and exclusion criteria were developed based on established systematic review methodology, focusing on publication quality, study design, population, intervention, comparison and outcome (Eriksen & Frandsen, 2018; Meline, 2006) (see Table 1). Specific patient populations, including children, people with traumatic brain injury and pregnant women, were excluded in acknowledgement that the mechanism of coagulopathy in these groups may be different to that of the general adult population (Attard et al., 2014; Conti et al., 2016; Leeper et al., 2018; Moon and Sappenfield, 2016).

**Table 1.** Inclusion and exclusion criteria.

**Inclusion criteria**	Publication type: original research published in peer-reviewed journals Language: restricted to English Study design: randomised controlled trials, non-randomised experimental design, surveys, interviews and focus groups. Studies within past 10 years Population: patients with traumatic injuries involved with pre-hospital clinicians Intervention: recognising ATC in the pre-hospital setting Comparison: various trauma presentations (blunt or penetrating trauma) Outcome: benefit to ATC patient referral
**Exclusion criteria**	Publication type: literature reviews, systematic reviews, editorials, comments, conference presentations and book chapters Study design: case reports and studies before 2009 Population: any patient not in a pre-hospital setting, such as in a hospital or laboratory, and studies that focused on animals Intervention: ATC assessment initiated on arrival at hospital in the emergency department Comparison: non-trauma presentations, pregnancy, children or traumatic brain injury Outcome: coagulopathy not the primary aim

**Figure 1:**
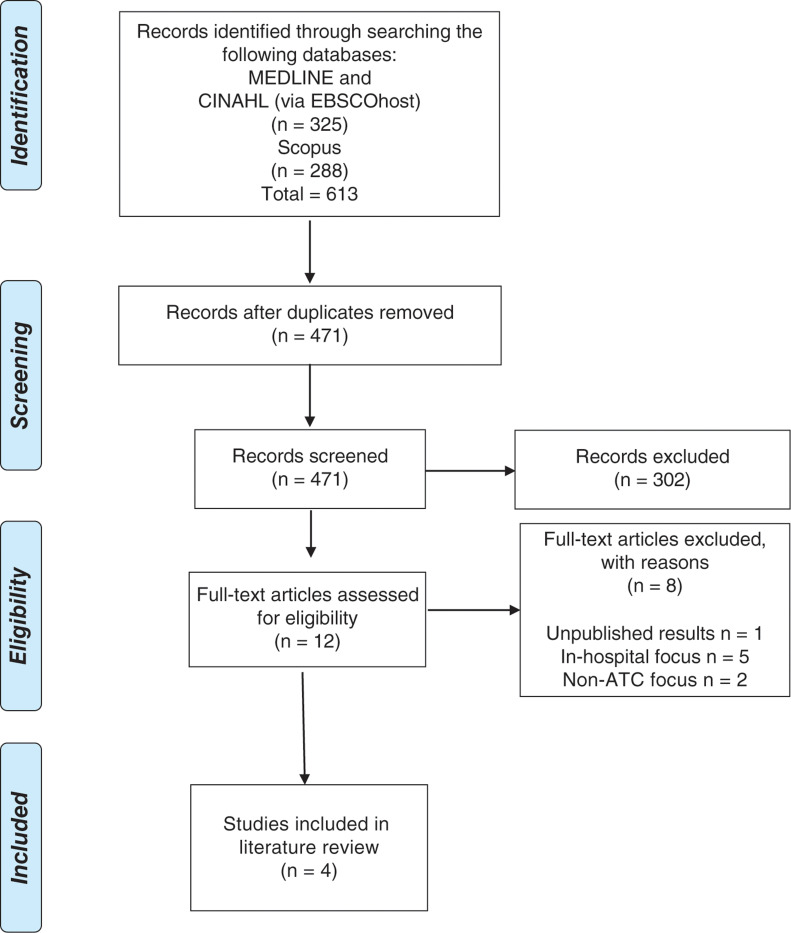
Figure 1. PRISMA flow diagram. Adapted from Moher et al. (2009).

A PRISMA method was used to identify and report on eligible articles (Moher et al., 2009) (see Figure 1). The appropriate Critical Appraisals Skills Programme (CASP) (2017) checklist was used to assess and appraise the quality of the research. Themes were then identified from the results of each study jointly by the authors in a concept matrix.

## Results

The initial search generated 613 results. Once duplicates had been removed, 471 records were screened, resulting in 12 eligible for full-text review. Eight articles were further excluded due to ongoing research with unpublished results (Tonglet et al., 2019), a focus on assessing variation in pre- and in-hospital coagulation (Floccard et al., 2012; Theusinger et al., 2015), retrospective exploration of traumatic induced coagulopathy from medical treatment rather than ATC (David et al., 2017), using decision tools for blood transfusion rather than ATC recognition (Tonglet et al., 2017) and ATC point-of-care testing commencing in hospital (Gauss et al., 2014; Goodman et al., 2015; Mistral et al., 2017). Four articles were found suitable for discussion on themes identified as prediction tools and point-of-care testing (see Table 2).

**Table 2.** Findings from studies.

Study	Purpose	Method	Time frame	Sample size	Investigative measures	Relevant findings and themes
**Mitra et al. (2011)**	Develop a tool that can identify pre-hospital ATC	Quantitative retrospective study Prospective validation 2nd stage Australia	August 2006 – July 2008 2nd stage January–December 2009	Derivation set: 1680; 151 identified with ATC Validation set (2nd stage): 1225; 100 noted as coagulopathic	Compared vital signs between major trauma patients who had ATC and those who did not The second stage examined the score’s prediction	Theme: Pre-hospital prediction tool The Coagulopathy of Severe Trauma (COAST) score was devised from significant vital signs associated with ATC (entrapment (p < 0.001), temperature (p < 0.001), systolic blood pressure (p < 0.001), abdominal or pelvic injury (p < 0.001) and pre-hospital needle thoracocentesis (p < 0.001)) A score ≥ 3 had 60% sensitivity and 96.4% specificity
**Tonglet et al. (2014)**	Distinguish patients who need damage control resuscitation from those who do not in major trauma	Quantitative prospective study Belgium	January 2012 – June 2013	82	Paramedics and pre-hospital doctors were trained in calculating the Trauma Induced Coagulopathy Clinical Score (TICCS) TICCS effectiveness was assessed in discriminating major trauma patients who required damage control resuscitation	Theme: Pre-hospital prediction tool TICCS is easy to use by paramedics, and is rapid at identifying blunt trauma patients requiring damage control resuscitation. A threshold score of 10 provided the best sensitivity (100%, 95% CI: 0.92–1.0) and specificity (95.9%, 95% CI: 88.2–99.2) Pre-hospital TICCS evaluation should allow initiation of optimal care upon hospital admission
**Beynon et al. (2015)**	Investigate point-of-care coagulometry in the pre-hospital setting	Quantitative prospective study Germany	12 months	103 Trauma = 19	To test the validity and potential value of a point-of-care coagulometer in identifying coagulopathy in pre-hospital patients, and compare INR accuracy against hospital laboratory results	Theme: Point-of-care testing CoaguChek® XS Pro Point-of-care coagulometry is associated with substantial gain in time when assessing haemostasis in emergency patients compared with central laboratory results (median gain of time = 69 minutes) Point-of-care INR of > 1.3 identified all patients with coagulopathy
**Peltan et al. (2016)**	Develop and validate a pre-hospital prediction model for ATC	Two-part quantitative study Part one retrospective cohort; part two prospective validation cohort USA	2008–2012	The derivation cohort consisted of 1963 patients The validation cohort consisted of 285 patients	Develop the effectiveness of the PACT score Constructed from retrospective data identifying key elements of patients at risk of ATC, including: age, injury mechanism, pre-hospital shock index, GCS, pre-hospital CPR and ET intubation	Theme: prediction tools: the Prediction of Acute Coagulopathy of Trauma (PACT) score A PACT score cut-off at ≥ 196 maximised sensitivity (73.1%) and specificity (73.8%) Discrimination and calibration of the PACT score was improved relative to that of another ATC prediction model (COAST score)

## Discussion

### Pre-hospital prediction tools

Mitra et al. (2011) sought to develop a tool that can identify ATC in the pre-hospital setting. Based on a retrospective analysis of 1680 trauma patients’ pre-hospital vital signs and type of injury, Mitra et al. (2011) formulated the Coagulopathy of Severe Trauma (COAST) score, which was further validated within the study with a prospective comparison cohort of 1225 patients. The authors concluded that specific vital signs were more likely to be apparent in ATC, forming a score ranging from 0 to 7 (see Table 3). A score ≥ 3 was found to have 60% sensitivity and 96.4% specificity. Interestingly, the specificity of the COAST score rose as the score increased (100% when ≥ 5), and sensitivity increased as the COAST score decreased (100% ≥ 0).

**Table 3.** Coagulopathy of Severe Trauma (COAST) score (Mitra et al., 2011).

Variable	Value	Score
**Entrapment**	Yes	1
**Systolic blood pressure**	< 100 mmHg	1
	< 90 mmHg	2
**Temperature**	< 35°C	1
	< 32°C	2
**Chest decompression**	Yes	1
**Abdominal or pelvic content injury**	Yes	1
**Highest total**		7

Mitra et al. (2011) elected not to include other vital signs, including the Glasgow Coma Score (GCS) (p = 0.03) as part of COAST, possibly due to the need to conduct an additional value, which could complicate the scoring process. Further criticisms of the study are the decision to exclude chest trauma as part of the score (n = 119, p = 0.001), and the lack of penetrating trauma in either comparison group.

Indeed, in a similar study, Peltan et al. (2016) compared the COAST score with their derived Prediction of Acute Coagulopathy of Trauma (PACT) score, finding that the PACT was significantly better at determining patients at risk of ATC than COAST. However, a PACT score had only a moderate sensitivity and specificity (73.1% and 73.8% respectively). Moreover, the PACT score includes a complex scoring system, requiring a calculation of the GCS and the pre-hospital shock index, comprised of heart rate divided by systolic blood pressure, cardiopulmonary resuscitation and advanced airway, where points per variable are not equally weighted. It is unclear why the authors decided to include the shock index when systolic blood pressure was equally significant (p < 0.001) and easier to calculate. Potentially, it was due to the median initial pre-hospital systolic blood pressure being greater than 100 mmHg (119 mmHg) despite confirmed coagulopathy, in contradiction to existing literature. This observational relationship could be explored further in the pre-hospital setting, as it could be that a cohort of patients could be suffering from ATC despite being normotensive.

Additionally, Peltan et al. (2016) did not assess pre-hospital temperature, which may have been different and more relevant when comparing with COAST, thus invalidating their claim that PACT is more discriminative in the pre-hospital setting. Further research would need to validate if PACT is suitable in the pre-hospital environment.

Tonglet et al. (2014) investigated pre-hospital application of the Trauma Induced Coagulopathy Clinical Score (TICCS). TICCS comprises three components with a score range of 0–18 (see Table 4). Tonglet et al. (2014) identified that paramedics and doctors who scored TICCS ≥ 10 significantly determined severe ATC patients requiring damage control resuscitation (sensitivity 100% CI: 53.9–100, specificity 95.9% CI: 88.2–99.2, p = 0.0011), a treatment strategy consisting of haemorrhage cessation, permissive hypotension, avoiding unnecessary fluid administration and targeting coagulopathy. The small sample (n = 82) – in which only eight required damage control resuscitation – as well as poor heterogeneity (78.1% of the group were male) and lack of reporting the mechanism of injury mean these findings may not be generalisable. Considering that TICCS requires assessment of trauma severity and extent of tissue injury, this type of information would have been useful in assessing the reliability of the tool.

**Table 4.** Trauma Induced Coagulopathy Clinical Score (TICCS) (Tonglet et al., 2014).

General trauma severity – 2 points	Judged to be in critical condition including: General severity of trauma Mechanism of injury Airway and breathing examinations Glasgow Coma Scale reduced
Systolic blood pressure – 5 points	Below 90 mmHg at least once
Extent of tissue injury – 11 points	+1 head and neck region +1 for each limb +2 torso +2 abdominal +2 pelvis

Although the three studies share different findings, some common themes within the results and tools emerge. Firstly, vital signs such as GCS and systolic blood pressure, and trauma to the chest, abdomen and pelvis, are key pre-hospital readings that could determine ATC. Unsurprisingly, these are all within the first two steps of the UK pre-hospital major trauma triage tool clinical guidelines (Joint Royal Colleges Ambulance Liaison Committee (JRCALC), 2019) (see Table 5).

**Table 5.** UK pre-hospital major trauma triage tool – steps 1 and 2.

**Step 1:** Physiological	GCS < 14 Systolic blood pressure < 90 mmHg
**Step 2:** Anatomical	Penetrating to head/neck/torso/limbs proximal to elbow/knee Chest injury with altered physiology Two proximal long bone fractures Crushed/degloved/mangled extremity Amputation proximal to wrist/ankle Pelvic fractures Open or depressed skull fracture Sensory or motor deficit (new onset following trauma)

Nevertheless, the linearity of the pre-hospital major trauma tool could be omitting patients with ATC, particularly in the presence of polytrauma, where ATC is more likely (Davenport, 2013). Moreover, the existing pre-hospital major trauma tool renders it difficult for pre-hospital clinicians to express through the tool which injury may be a more significant priority until arrival at the MTC. Yet, in identifying ATC at the earliest opportunity, a paramedic could highlight the need to accelerate blood transfusion triggers at hospital (Brohi et al., 2019). This concept is not new among physician-led pre-hospital teams, where studies by Weaver et al. (2016) and Reed et al. (2016) have illustrated how a pre-hospital ‘code red’ protocol for UK major trauma patients to receive blood transfusion on arrival at hospital improves treatment timeliness and survival. Importantly, physician-led resources remain limited in the pre-hospital setting, where timeliness to definitive care, such as an MTC, remains the priority in major trauma and ATC (Cohen & Christie, 2017; Thompson et al., 2017).

However, ‘code red’ already comprises similar criteria to TICCS, including systolic blood pressure < 90 mmHg and suspected or active bleeding (Weaver et al., 2016), where TICCS has been further evidenced to reduce mortality in trauma patients when pre-hospital clinicians alert hospitals, and to improve timely preparation of blood products (Tonglet et al., 2017). Therefore, future studies should explore whether paramedics can utilise screening tools to highlight individuals at risk of ATC, and implement specific care pathways such as ‘code red’, improving care and patient outcomes from ATC.

### Point-of-care testing

The second theme identified the possible use of a handheld coagulometer in detecting coagulopathy at the point of pre-hospital care. Beynon et al. (2015) used a handheld point-of-care device (Coaguchek®) to compare the pre-hospital international normalised ratio (INR) with hospital laboratory values, followed by a brief survey of the pre-hospital physician. Although 19 of the samples were specifically recognised as trauma, Beynon et al. (2015) identified that all patients with coagulopathy had an INR > 1.3 (sensitivity 100%, specificity 98.7%), where point-of-care INR significantly correlated with laboratory INR (0.68, p < 0.0001). A median time gain of 69 minutes in using the device to detect coagulopathy was highlighted as beneficial for rapid recognition and treatment escalation. However, 42% of pre-hospital physicians felt that the value of point-of-care INR assessment was low, and no physicians would consider any further treatment at the scene.

Furthermore, the point-of-care assessment relies on a blood sample within two minutes of venepuncture, and cannot function in temperatures lower than 5°C or higher than 35°C. Certainly in the UK, such a device would be unreliable during cold winter months. Another confounding factor would be if an individual is on anti-coagulation medication, requiring enhanced knowledge of the threshold of INR, the role of anticoagulant reversal agents and when delay or aggressive treatment is appropriate (Mullins et al., 2018). Such decisions require expertise currently beyond the scope of a pre-hospital paramedic. Additionally, utilisation of such a device in a difficult situation such as major trauma may not be feasible, especially if multiple treatments or rapid extrication are required, where acquiring a blood sample in less than two minutes may not be a priority.

Furthermore, point-of-care devices applied in the emergency department to identify ATC have been shown to have weak correlation with laboratory values (Goodman et al., 2015; Mistral et al., 2017). While NICE (2016) recommends early INR monitoring in ATC, currently this practice remains poorly evidenced and has apparently limited use in improving ATC care or referral in the pre-hospital realm.

### Limitations

The paucity of literature, and lack of repeated studies using the same tool within the pre-hospital setting, render very limited evidence. One study investigated point-of-care, with a very small sample that limits any comparison or accurate conclusion. Lastly, studies focused on various countries that may not be representative of the UK population or paramedic practice.

## Conclusion

This literature review sought to establish if screening tools could identify ATC in the pre-hospital setting, which could be utilised by paramedics. While studies are few, with various small cohorts across the world and of limited evidence, there is potential for tools such as the COAST score or TICCS to predict ATC, and initiate a care plan such as a code red for blood products.

The authors recommend that if this were to be investigated further, it should be incorporated within the pre-hospital major trauma triage tool, such that if multiple steps are flagged between steps 1 and 2, an ATC protocol is initiated – or be utilised by experienced critical care paramedics as part of a holistic management plan.

### Key points

- Recognition and management of acute traumatic coagulopathy (ATC) need to improve in the UK. Paramedics are in an optimal position to identify ATC, to initiate damage control resuscitation and to alert receiving hospitals to prepare blood products.
- Pre-hospital prediction tools such as the COAST score and TICCS show promise in identifying ATC early, and could be used to initiate an early blood transfusion protocol at the awaiting Major Trauma Centre. This needs to be researched further to see if a particular score or threshold is optimal.
- Future research could investigate if ATC screening tools can be adapted into the pre-hospital major trauma tool to flag suspected ATC.
- Point-of-care testing is unlikely to be beneficial in the pre-hospital setting.

## Author contributions

SR co-wrote the Method section, wrote the Results and Discussion sections and did the overall review and writing of the article. JK wrote the Introduction section, co-wrote the Method section, co-identified themes and reviewed the Discussion section. SR acts as the guarantor for this article.

## Conflict of interest

None declared.

## Funding

None.

## References

[bibr_1] Attard C. Straaten T. Karlaftis V. Monagle P. & Ignjatovic V. (2014). Developmental haemostasis: Age-specific differences in the quantity of hemostatic proteins: Reply to a rebuttal. *Journal of Thrombosis and Haemostasis*, 12(2), 286–286. 24283702 10.1111/jth.12462

[bibr_2] Beynon C. Erk A. G. Potzy A. Mohr S. & Popp E. (2015). Point of care coagulometry in prehospital emergency care: An observational study. *Scandinavian Journal of Trauma, Resuscitation and Emergency Medicine*, 23(1), 58–64. 26260487 10.1186/s13049-015-0139-6 PMC4542099

[bibr_3] Brohi K. & Eaglestone S. (2017). Traumatic coagulopathy and massive transfusion: Improving outcomes and saving blood. *Programme Grants Appl Res.*, 5(17), 1–74. 29188703

[bibr_4] Brohi K. Gruen R. L. & Holcomb J. B. (2019). Why are bleeding trauma patients still dying? *Intensive Care Medicine*, 45(5), 709–711. 30741331 10.1007/s00134-019-05560-x

[bibr_5] Cohen M. J. & Christie S. A. (2017). Coagulopathy of trauma. *Critical Care Clinics*, 33(1), 101–118. 27894491 10.1016/j.ccc.2016.08.003

[bibr_6] Conti B. Villacin M. K. & Simmons J. W. (2016). Trauma anesthesia for traumatic brain injury. *Current Anesthesiology Reports*, 6(1), 95–101.

[bibr_7] Critical Appraisal Skills Programme (CASP). (2017). *CASP case control study checklist*. Retrieved November 2, 2019, from http://docs.wixstatic.com/ugd/dded87_afbfc99848f64537a53826e1f5b30b5c.pdf.

[bibr_8] Davenport R. (2013). Pathogenesis of acute traumatic coagulopathy. *Transfusion*, 53, 23S–27S. 23301969 10.1111/trf.12032

[bibr_9] David J. S. Voiglio E. J. Cesareo E. Vassal O. Decullier E. Gueugniaud P. Y. Peyrefitte S. & Tazarourte K. (2017). Prehospital parameters can help to predict coagulopathy and massive transfusion in trauma patients. *Vox Sanguinis*, 112(6), 557–566. 28612932 10.1111/vox.12545

[bibr_10] Durham M. (2017). Paramedic accuracy and confidence with a trauma triage algorithm: A cross-sectional survey. *British Paramedic Journal*, 1(4), 1–7.

[bibr_11] Eriksen M. B. & Frandsen T. F. (2018). The impact of patient, intervention, comparison, outcome (PICO) as a search strategy tool on literature search quality: A systematic review. *Journal of the Medical Library Association: JMLA*, 106(4), 420–431. 30271283 10.5195/jmla.2018.345 PMC6148624

[bibr_12] Floccard B. Rugeri L. Faure A. Saint Denis M. Boyle E. M. Peguet O. Levrat A. Guillaume C. Marcotte G. Vulliez A. & Hautin E. (2012). Early coagulopathy in trauma patients: An on-scene and hospital admission study. *Injury*, 43(1), 26–32. 21112053 10.1016/j.injury.2010.11.003

[bibr_13] Gauss T. Hamada S. Jurcisin I. Dahmani S. Boudaoud L. Mantz J. & Paugam-Burtz C. (2014). Limits of agreement between measures obtained from standard laboratory and the point-of-care device Hemochron Signature Elite® during acute haemorrhage. *British Journal of Anaesthesia*, 112(3), 514–520. 24335551 10.1093/bja/aet384

[bibr_14] Goodman M. D. Makley A. T. Hanseman D. J. Pritts T. A. & Robinson B. R. (2015). All the bang without the bucks: Defining essential point-of-care testing for traumatic coagulopathy. *The Journal of Trauma and Acute Care Surgery*, 79(1), 117–124. 26091324 10.1097/TA.0000000000000691 PMC5558264

[bibr_15] Joint Royal Colleges Ambulance Liaison Committee (JRCALC), Association of Ambulance Chief Executives. (2019). *JRCALC clinical guidelines 2019*. Class Professional Publishing.

[bibr_16] Leeper C. M. Neal M. D. McKenna C. Billiar T. & Gaines B. A. (2018). Principal component analysis of coagulation assays in severely injured children. *Surgery*, 163(4), 827–831. 29248181 10.1016/j.surg.2017.09.031

[bibr_17] Meline T. (2006). Selecting studies for systematic review: Inclusion and exclusion criteria. *Contemporary Issues in Communication Science and Disorders*, 33, 21–27.

[bibr_18] Mistral T. Boué Y. Bosson J. L. Manhes P. Greze J. Brun J. Albaladejo P. Payen J. F. & Bouzat P. (2017). Performance of point-of-care international normalized ratio measurement to diagnose trauma-induced coagulopathy. *Scandinavian Journal of Trauma, Resuscitation and Emergency Medicine*, 25(1), 59–66. 28637514 10.1186/s13049-017-0404-y PMC5480161

[bibr_19] Mitra B. Cameron P. A. Mori A. Maini A. Fitzgerald M. Paul E. & Street A. (2011) Early prediction of acute traumatic coagulopathy. *Resuscitation*, 82(9), 1208–1213. 21600687 10.1016/j.resuscitation.2011.04.007

[bibr_20] Moher D. Liberati A. Tetzlaff J. Altman D. G. & The Prisma Group. (2009) Preferred reporting items for systematic reviews and meta-analyses: The PRISMA statement. *PLoS Medicine*, 6(7), 1–6. 10.1371/journal.pmed.1000097 PMC2707599 19621072

[bibr_21] Moon T. S. & Sappenfield J. (2016). Anesthetic management and challenges in the pregnant patient. *Current Anesthesiology Reports*, 6(1), 89–94.

[bibr_22] Mullins B. Akehurst H. Slattery D. & Chesser T. (2018). Should surgery be delayed in patients taking direct oral anticoagulants who suffer a hip fracture? A retrospective, case-controlled observational study at a UK major trauma centre. *BMJ Open*, 8(4), 1–7. 10.1136/bmjopen-2017-020625 PMC5931299 29705761

[bibr_23] National Institute for Health and Care Excellence (NICE). (2016). Major trauma: Assessment and initial management. Retrieved November 3, 2019, from https://www.nice.org.uk/guidance/ng39/chapter/recommendations-for-research#1-point-ofcare-coagulation-testing. 26913320

[bibr_24] Olaussen A. Semple W. Oteir A. Todd P. & Williams B. (2017). Paramedic literature search filters: Optimised for clinicians and academics. *BMC Medical Informatics and Decision Making*, 17(1), 146–152. 29020951 10.1186/s12911-017-0544-z PMC5637081

[bibr_25] Peltan I. D. Rowhani-Rahbar A. Vusse L. K. V. Caldwell E. Rea T. D. Maier R. V. & Watkins T. R. (2016). Development and validation of a prehospital prediction model for acute traumatic coagulopathy. *Critical Care*, 20(1), 371–381. 27846895 10.1186/s13054-016-1541-9 PMC5111191

[bibr_26] Reed M. J. Glover A. Byrne L. Donald M. McMahon N. Hughes N. Littlewood N. K. Garrett J. Innes C. McGarvey M. Hazra E. & Rawlinson S. (2016). Experience of implementing a national pre-hospital code red bleeding protocol in Scotland. *Injury*, 48(1), 41–46. 27641222 10.1016/j.injury.2016.09.020

[bibr_27] Theusinger O. M. Baulig W. Seifert B. Müller S. M. Mariotti S. & Spahn D. R. (2015) Changes in coagulation in standard laboratory tests and ROTEM in trauma patients between on-scene and arrival in the emergency department. *Anesthesia & Analgesia*, 120(3), 627–635. 25545751 10.1213/ANE.0000000000000561

[bibr_28] Thompson L. Hill M. Davies C. Shaw G. & Kiernan M. D. (2017). Identifying pre-hospital factors associated with outcome for major trauma patients in a regional trauma network: An exploratory study. *Scandinavian Journal of Trauma, Resuscitation and Emergency Medicine*, 25(1), 1–8. 28835283 10.1186/s13049-017-0419-4 PMC5569481

[bibr_29] Tonglet M. D’Orio V. Moens D. Lens F. X. Alves J. Thoma M. Kreps B. Youatou Towo P. Betz R. Piazza J. & Szecel J. (2019). Impact of a prehospital discrimination between trauma patients with or without early acute coagulopathy of trauma and the need for damage control resuscitation: Rationale and design of a multicenter randomized phase II trial. *Acta Chirurgica Belgica*, 119(2), 88–94. 29745298 10.1080/00015458.2018.1470276

[bibr_30] Tonglet M. Lefering R. Minon J. M. Ghuysen A. D’Orio V. Hildebrand F. Pape H. C. & Horst K. (2017). Prehospital identification of trauma patients requiring transfusion: results of a retrospective study evaluating the use of the Trauma Induced Coagulopathy Clinical Score (TICCS) in 33,385 patients from the TraumaRegister DGU®. *Acta Chirurgica Belgica*, 117(6), 385–390. 28639537 10.1080/00015458.2017.1341148

[bibr_31] Tonglet M. Minon J. M. Seidel L. Poplavsky J. L. & Vergnion M. (2014). Prehospital identification of trauma patients with early acute coagulopathy and massive bleeding: Results of a prospective noninterventional clinical trial evaluating the Trauma Induced Coagulopathy Clinical Score (TICCS). *Critical Care*, 18(6), 648. 25425230 10.1186/s13054-014-0648-0 PMC4279963

[bibr_32] Trauma Audit and Research Network. (2017). *England & Wales: Major trauma in older people – 2017*. The Trauma and Audit Research Network.

[bibr_33] Weaver A. E. Hunter-Dunn C. Lyon R. M. Lockey D. & Krogh C. L. (2016). The effectiveness of a ‘code red’ transfusion request policy initiated by pre-hospital physicians. *Injury*, 47(1), 3–6. 26239421 10.1016/j.injury.2015.06.023

